# Prevalence of methicillin-resistant *Staphylococcus aureus* (MRSA) in nasal swab specimens obtained from hospitalized patients and healthcare workers in a Belgrade hospital

**DOI:** 10.1186/1753-6561-5-S6-P16

**Published:** 2011-06-29

**Authors:** B Jovanovic, Ivana Cirkovic

**Affiliations:** 1 Hospital Epidemiology, Clinical Center of Serbia, Belgrade, Serbia

## Introduction / objectives

The aim of the present study was to provide the analysis of carriage of MRSA in hospitalized patients and HCWs in the largest healthcare facility in Serbia.

## Methods

Nasal swab were taken from 195 hospitalized and 105 HCWs at the Clinical Center of Serbia in Belgrade. Each swab was inoculated directly onto MRSA-ID agar (bioMérieux, France). All inoculated solid media were incubated at 35°C and read after 24 h and 48 h of incubation. Identification of isolates was confirmed by PCR for *nuc* and *mec*A gene. Susceptibility to antibiotics was performed by disk diffusion method in accordance to the CLSI recommendations. Determination of SCC*mec* types was done by previously described PCR protocol.

## Results

Among 195 hospitalized patients and 105 HCWs, 23 (11.8%) and 8 (7.6%) respectively were colonized MRSA*.* All tested MRSA strains were susceptible to fusidic acid, trimethoprim/sulfamethoxazole, vancomycin, linezolid, pristinamycin and mupirocin, while 27 (87.1%) were resistant to gentamicin, 28 (90.3%) to kanamycin, 27 (87.1%) to tobramycin, 17 (54.8%) to erythromycin, 17 (54.8%) to clindamycin, 25 (80.6%) to ciprofloxacin, 3 (9.7%) to rifampin, 4 (12.9%) to tetracycline and 5 (16.1%) to chloramphenicol. Among MRSA strains isolated in this study, 6 (19.4%) strains could be classified as CA-MRSA, because they were SCC*mec* type IV or V and most of them were susceptible to all tested antibiotics except beta-lactams. The remaining 25 (80.6%) MRSA strains had characteristics of HA-MRSA, they were SCC*mec* I or III, and all were resistant to different antibiotics beside beta-lactams.

## Conclusion

Carriage of MRSA among hospitalized patients and HCWs was determined to be high, 10.3%. Carriage was higher in hospitalized patients than in HCWs. Most of the isolated strains were HA-MRSA.

## Disclosure of interest

None declared.

